# Acute and Post-Acute Neurological Complications of COVID-19

**DOI:** 10.3390/neurolint13010010

**Published:** 2021-03-09

**Authors:** Ali Al-Ramadan, Omar Rabab’h, Jawad Shah, Abeer Gharaibeh

**Affiliations:** 1 Insight Research Institute, Flint, MI 48507, USA; medsali@umich.edu (A.A.-R.); medsomar@umich.edu (O.R.); Shah.research@iinn.com (J.S.); 2 Center for Cognition and Neuroethics, University of Michigan-Flint, Flint, MI 48502, USA; 3 Insight Research Center, Insight Institute of Neurosurgery & Neuroscience, Flint, MI 48507, USA; 4 Department of Medicine, Michigan State University, East Lansing, MI 48824, USA; 5 Insight Surgical Hospital, Warren, MI 48091, USA

**Keywords:** SARS-CoV-2, COVID-19, neurological complications, cerebrovascular complications, encephalopathy, demyelinating disorders, nervous system invasion, molecular mimicry, COVID-19 treatments, post-acute COVID-19 syndrome

## Abstract

Coronavirus disease 2019 (COVID-19) is an emerging global health emergency caused by the novel coronavirus, severe acute respiratory syndrome coronavirus 2 (SARS-CoV-2). The global outbreak of SARS-CoV-2 infection has been declared a global pandemic by the World Health Organization (WHO). The clinical presentation of SARS-CoV-2 infection depends on the severity of the disease and may range from an asymptomatic infection to a severe and lethal illness. Fever, cough, and shortness of breath are among the most common symptoms associated with SARS-CoV-2 infection. Accumulating evidence indicates that COVID-19 patients commonly develop neurological symptoms, such as headache, altered mental status, anosmia, and myalgia. In this comprehensive literature review, we have summarized the most common neurological complications and reported neurological case studies associated with COVID-19, and neurological side effects associated with COVID-19 treatments. Additionally, the post-acute COVID-19 syndrome and long-term neurological complications were discussed. We also explained the proposed mechanisms that are involved in the pathogenesis of these neurological complications.

## 1. Introduction

Coronavirus disease 2019 (COVID-19), which is caused by the novel coronavirus, severe acute respiratory syndrome coronavirus 2 (SARS-CoV-2), is an emerging pandemic that was first described in December 2019 in China [1,2]. The SARS-CoV-2 viral infection has spread all over the world resulting in more than 99 million confirmed cases, and two million deaths globally by January 2021 [3]. The mortality rate associated with COVID-19 varies among different countries, which can be attributed, in part, to the median age of the affected population and the availability of critical resources needed to treat the patients [4].

The clinical manifestations of the COVID-19 are highly variable, with 40–45% of COVID-19 positive cases being asymptomatic [5]. A study by Zhang and colleagues reported the clinical characteristics of 140 hospitalized COVID-19 patients. In this study, the male to female ratio was 1:1, and the median age was 57 years. The most common observed symptoms were fever, cough, and fatigue. Among these patients, the most common co-morbidities were hypertension and diabetes mellitus [6]. Another systematic review by Fu and colleagues investigated the key clinical characteristics of COVID-19 patients. The report summarized a total of 43 studies and included 3600 patients. The most common clinical features associated with COVID- 19 included fever (83.3%), cough (60.3%), fatigue (38.0%), sputum production (30%), shortness of breath 30%), myalgia (30%), sore throat (16.7% and 11.2% in critical and non-critical cases, respectively), and headache (11.3% and 11.9% in critical and non-critical cases, respectively). However, the study demonstrated that the majority of patients with COVID-19 were asymptomatic [7]. These findings were confirmed by another retrospective study by Wang and colleagues, which investigated non-critically ill patients with COVID-19 [8]. In a prospective cohort study by Cummings and colleagues, a total of 1150 patients with confirmed COVID-19 and a median age of 62 years were included. Among this cohort of patients, 22% were critically ill, and 67% of the critically ill patients were men. The most common reported symptoms were shortness of breath (74%), fever (71%), cough (66%) and myalgia (26%). Thirty nine percent of these patients died [9].

According to many reports and studies, the newly emerging SARS-CoV-2 virus that causes COVID-19 is associated with neurological complications. These complications could impact both the central and peripheral nervous systems. The neurological manifestations could be non-specific such as headache, altered mental status, and myalgia or more specific diseases and syndromes which require immediate medical attention [10,11,12,13].

## 2. Pathophysiology and Mechanisms

SARS-CoV-2 virus is a member of the Coronaviridae, which is a large family of single-stranded RNA viruses [(+) ssRNA] that can be found in a variety of animal species [14]. SARS-CoV-2 membrane is characterized by the presence of the spike (S) glycoprotein, which facilitates entry into cells. SARS-CoV-2 is mainly transmitted by coughing and sneezing through respiratory droplets. It invades the respiratory system through attaching to the respiratory epithelium and it enters the cells through binding to the angiotensin-converting enzyme 2 (ACE2) receptors. The viral incubation period varies among patients, and ranges from three to seven days [14]. Based on the mode of viral transmission and entry, SARS-CoV-2 mainly targets the respiratory system inducing pathological pulmonary changes, such as alveolar damage, hyaline membrane formation, interstitial fibrosis, and immune infiltration [15].

The exact mechanism by which the cells die is not clear yet but could be due to a combination of direct viral cytopathic effects and hyperactivation of the host immune response [16]. The immune response to SARS-CoV-2 is a double-edged sword; the immune cells that are recruited to the infection site can inflict further damage to the infected tissues [16]. The induction of a cytokine storm through the release of proinflammatory cytokines has been implicated in the pathological complications associated with COVID-19 [17].

The mechanisms of SARS-CoV-2- associated neurological manifestations are still not fully understood. Several mechanisms have been proposed to explain SARS-CoV-2-induced neurological disorders. Neurological infections by coronaviruses have been previously described in patients with Severe Acute Respiratory Syndrome (SARS), which is caused by SARS-CoV virus, and Middle East Respiratory Syndrome (MERS), which is caused by MERS-CoV. The severity and consequences of these infections vary among patients [18]. The evaluation of SARS-CoV-2 invasion into the cerebrospinal fluid (CSF) is very limited and positive results are rare as well [19]. A systematic review by Lewis and colleagues found that 6% of the patients who had a CSF analysis had a positive SARS-CoV-2 in their CSF [20]. CSF cell count was increased in 43% of fatal cases, 25.7% of severe cases, and 29.4% of non-severe cases [20]. The majority of those patients have neurological symptoms related to the central nervous system (CNS). Another systematic review by Tandon and colleagues assessed CSF protein levels in COVID-19 patients [21]. Elevated CSF proteins were found to be the most common CSF finding. They found that patients with fatal COVID-19 had higher levels of proteins in their CSF (100%) with an average of 61.28 mg/dl in comparison to nan-fatal cases (65%) with an average of 56.73 mg/dl. CSF protein levels were also found to be elevated in 74.5% of patients with non-severe COVID-19 and 68.6% in those with a severe COVID-19 infection.

Potential direct viral invasion of the nervous system is supported by many observations [21,22,23,24,25,26]. First, ACE2 receptors, which are the entry site for the SARS-CoV-2, are expressed in the brain tissues [23]. Second, SARS-CoV-2 shares similarities with SARS-CoV which has been shown previously to invade the nervous system based on preclinical studies and postmortem analysis [24]. Third, postmortem analysis of a patient with neurological manifestation detected SARS-CoV-2 in the frontal lobe [25]. Fourth, meningitis and encephalitis were reported as isolated presentations concomitant with SARS-CoV-2 detection in the CSF in certain cases [21,22].

On the other hand, some studies showed that SARS-CoV-2 virus was not detected in the CNS [27,28,29], and some suggested that positive detection of the virus could be blood–brain barrier breakdown or presence of in situ viral particles [27,29]. Brann and colleagues carried out a study to identify the cells that are susceptible to SARS-CoV-2 infection and found that the supporting cells in the olfactory epithelium express comparable levels of the virus entry receptors similar to those found in the lung. Notably, the expression of those receptors was not detected in olfactory sensory neurons and olfactory bulbs. Based on these findings, the authors suggest that cytokine release by infected supporting cells may affect olfactory function. Damaging of supporting cells and decreasing blood supply caused by vascular damage could ultimately affect the smell perception [30].

The immune-mediated role in COVID-19 pathogenesis is also noted in many cases. Many patients with severe COVID-19 disease experience a surge of proinflammatory cytokines, a condition known as a cytokine storm [31]. Cytokine storms can cause damage to the blood-brain- barrier, and their potential role in neurological complications has been documented, specifically, in acute necrotizing encephalopathy cases [32,33]. Cytokine storm could also contribute to a form of headache that appears on the 7th–10th day of clinical onset of COVID-19 illness [34]. The role of immune system hyperactivation is also supported by the response of Guillain–Barré syndrome cases to immunoglobulin therapy and the presence of GD1b-IgG in a patient with Miller Fisher syndrome [35,36,37].

A possible explanation was also suggested for such immune response is the molecular mimicry [38]. In molecular mimicry, a foreign antigen can induce immune cells against self-antigen caused by sequence similarity between foreign and self-peptides [39]. Molecular mimicry is involved in many diseases such as multiple sclerosis and Guillain–Barré syndrome [39]. Autoantibodies have been detected in a number of patients with COVID-19 [40,41,42]. It is believed that these autoantibodies may contribute to the severity and complications of COVID-19 [41,42]. The role of autoantibodies in neurological complications was first suggested by the occurrence of Guillain–Barré syndrome in COVID-19 patients [38]. Lucchese and Flöel conducted a study and searched for the presence of peptide sequence similarity between SARS- CoV-2 and human self- antigens associated with Guillain–Barré syndrome. They found a shared immunogenic sequence between SARS-CoV-2 and HSP90B, HSP90B2 and HSP60 which are heat shock proteins [38]. Autoantibodies against heat shock proteins are involved in many neurological diseases including Guillain–Barré syndrome which support their hypothesis [43]. Recently, Franke and colleagues found a high frequency of autoantibodies against broad epitopes of the nervous system in critically ill patients with unexplained neurological complications [44]. These autoantibodies were against known neuronal proteins like Yo or NMDA receptors and others unknown proteins against endothelial cells, astrocytes and neuropils of basal ganglia, hippocampus, and olfactory bulbs [44]. The autoantibodies were also reported in two cases of encephalitis and in other patients with neurological symptoms [45,46,47]. However, the pathological role of autoantibodies needs to be determined. Interestingly, there is evidence of immune compartmentalization in the CNS of COVID-19 patients. Scientists found a specific CSF immunological alteration where there was elevated IL-12 and IL-1b in the CSF but not in the plasma of COVID-19 patients with neurological complications. Moreover, SARS-CoV-2 antibody profiles differ between CSF and plasma of COVID-19 patients [47]. Schiaffino and colleagues utilized indirect immunofluorescent technique to screen for autoantibodies in COVID-19 patients. The autoantigens that were examined were found to be non-organ specific. Additionally, a district immunofluorescence pattern specific for COVID-19 was observed. The presence of autoantibodies was also associated with higher incidence of neurological and thrombotic complications [48]. Mohkhedkar and colleagues used computational methods to identify the proteins that may act as autoantigens in COVID-19 patients with neurological complications. Searching for homology sequence between human proteins and SARS-CoV-2, they identified four candidate proteins. These are heat shock protein A5 (HSPA5), titin, Ryanodine receptor 2 (RYR2) which share homology sequence with spike proteins and heat shock protein 90 alpha family class B member 1 (HSP90AB1) [49].

Another factor that plays a role in neurological complications associated with COVID-19 is related to the severity of the illness. Coagulation abnormalities seen in cases of COVID-19 may precipitate strokes observed in COVID-19 patients [50]. Patients with severe COVID-19 illness develop many complications that could aggravate or contribute to the observed neurological complications. Since the brain is highly sensitive to hypoxia, prolonged COVID-19-mediated hypoxia could cause serious complications [51,52]. Systemic disturbances such as hypoxia [51] and electrolyte changes [53] that were seen in patients with COVID-19 may precipitate altered mental status [54] This is also supported by a study was done by Solomon and colleagues which showed acute hypoxic changes on histopathological examination of brain tissue of patients died within 32 days of the onset of COVID-19 infection [30]. However, Weyhern and colleagues performed histopathological examination on a series of six autopsies and found diffuse petechial hemorrhage in brains along with lymphocytic encephalitis and meningitis. Many territories in brains such as dorsal motor nuclei of the vagus nerve, cranial nerve V, nucleus tractus solitarii, dorsal raphe nuclei, and fasciculus longitudinalis medialis showed neuronal cell loss and axonal degeneration. Importantly, these findings were not consistent with hypoxic patterns [55].

The complexity and overlapping of neuropathological changes were best demonstrated by Reichard and colleagues [56]. They reported interesting neuropathological findings in a patient died from COVID-19. They found a characteristic subcortical white pathology with axonal loss, macrophage infiltration and perivascular acute disseminated encephalomyelitis (ADEM)-like pattern. The deep grey nuclei of the brainstem were not affected. Notably, the pathological features were not typical for viral and post-viral encephalitis and could not be explained by only global brain- hypoxic injury [56].

## 3. Neurological Complications

Several cases of neurological complications have been reported in patients with COVID-19. Although some complications present as non-specific symptoms, such as headache, fatigue, and altered mental status, certain neurological complications and diseases are thought to be directly linked to SARS-CoV-2 infection.

### 3.1. Non-Specific Neurological Complications

The incidence of non-specific neurological symptoms in patients with confirmed COVID-19 diagnosis has been reported by several studies. These symptoms include headache, altered mental status, dizziness, depressed level of consciousness, ageusia (loss of taste), anosmia (loss of smell), myalgia and fatigue [10,11,12,13]. A retrospective study by Mao and colleagues, which included 214 patients, demonstrated that 36.4% of these patients experienced neurological manifestations. Patients with severe clinical manifestations of COVID-19 were more likely to experience neurological symptoms compared with those with mild disease. Notably, CNS-related events were more common than peripheral nervous system (PNS) manifestations. About 24.8% of patients experienced CNS manifestations, such as headache, disturbance in consciousness, dizziness, acute cerebrovascular disease, ataxia, and seizures. Only 8.9% of the patients had PNS symptoms including impairment in taste, smell, and vision. Musculoskeletal symptoms were observed in 10.7% of the patients [10].

Non-specific neurological complications were also described in a report by Huang and colleagues, which investigated the clinical manifestations of COVID-19 in China through the analysis of data obtained from patients’ medical records and shared by the World Health Organization (WHO) and International Severe Acute Respiratory and Emerging Infection Consortium. The data confirmed the incidence of neurological complications in patients with COVID-19 including headache (8%), and myalgia or fatigue (44%) [11]. Another retrospective, single-center study by Chen and colleagues, which involved 99 patients with COVID-19, reported the observation of non-specific neurological manifestations including headache (8%), altered mental status (9%), and muscle ache (11%) [12].

A meta-analysis by Wang and colleagues reported that olfactory (35.7–85.6%) and gustatory (33.3–88.8%) disorders are common neurological manifestations of COVID19 [57]. In another study that included 417 patients with mild to moderate disease, Lechien and colleagues reported the incidence of olfactory and gustatory dysfunctions in 85.6% and 88.0% of the patients, respectively. Among these patients, 11.8% indicated that the olfactory dysfunction preceded other symptoms. Olfactory dysfunction was significantly associated with fever and gustatory symptoms, but not with rhinorrhea or nasal dysfunction [13]. Lechien and colleagues, in another large prospective study on mild COVID-19, found that females and younger patients were more frequently to report loss of smell [58].

Headache is also found to be a common symptom in COVID-19 patients. Wang and colleagues found in their systematic review that headache was the fourth common neurological symptom [57]. The Prevalence of headache was also reported to be as high as 70% in multicenter European study [58]. Additionally, headache was found to be an isolated symptom of COVID-19 in some cases [59]. A retrospective cohort study by Trigo and colleagues assessed the factors that are associated with the presence of headache in hospitalized COVID-19 patients and found that the frequency of headache was 23.7%. Interestingly headache was found to be an independent predictor of lower risk of mortality in hospitalized COVID-19 patients [60].

### 3.2. Specific Neurological Complications

Several neurological complications have been described in patients with COVID-19. Certain neurological symptoms were reported independently by several reports and studies, which suggest a potential real association with the pathogenesis of COVID-19.

#### 3.2.1. Cerebrovascular Complications

The arteries and veins, which form the cerebrovascular system, are essential for maintaining blood flow to and from the brain. The carotid arteries and jugular veins are the main blood vessels that form the backbone of the cerebrovascular circulation [61]. Occlusion or rupture of these blood vessels could interrupt blood perfusion of the brain, which could lead to stroke that manifests with neurologic deficits [61]. Several series of studies reported stroke in patients with COVID-19. A study reported five cases of COVID-19 patients, four males and one female under the age of 50, suffered a stroke. These observations may suggest a potential link between the pathogenesis of COVID-19 and stroke. The COVID-19 patients developed hemiplegia and had no medical history of any possible comorbidities. The patients included in this study presented with a wide range of disease severity ranging from asymptomatic infections to symptoms, such as fever, cough, and lethargy [62]. Beyrouti and colleagues described the characteristic features of ischemic stroke in six patients with COVID-19. Notably, all patients had large vessel occlusion, and most patients were found to have hypercoagulable state. Interestingly, one of the patients had a stroke as the initial presentation of COVID-19 [63].

In another case series, six patients from Italy with a median age of 69 years were reported to have had a stroke along with COVID-19. Ischemic stroke occurred in four patients and hemorrhagic stroke in two patients. Patients with severe COVID 19 developed strokes and had poor outcomes. In all cases but one, patients had severe pneumonia, and four patients developed organ failure. A total of five patients died and one patient suffered from severe neurological disabilities [64].

Other larger studies assessed stroke in patients with COVID-19. Li and colleagues analyzed 219 COVID-19 patients in a retrospective study. They found that 10 patients (4.6%) developed acute ischemic stroke and one patient (0.5%) had intracerebral hemorrhage. Patients were more likely to have hypertension and hypercoagulable status, and stroke indicated a poor prognostic marker [65]. Jain and colleagues investigated data from neuroimaging studies and the potential impact of neurological events on the outcomes of COVID-19 patients. They conducted a retrospective analysis of 3218 patients who were admitted to a tertiary hospital in New York city. The data demonstrated that the prevalence of acute stroke among COVD-19 patients was 1.1%. Moreover, stroke was the most common finding of neuroimaging studies and its incidence was an indicator of poor patients’ outcomes [66]. This result is further supported by another study by Merkler and colleagues which found that among 1916 patients who visited the emergency department or hospitalized patients, 31 patients (1.6%) developed acute ischemic stroke [67]. Additionally, Ntaios and colleagues assessed the severity of acute ischemic stroke in Global COVID-19 Stroke Registry and found that patients with COVID-19 had higher odds of developing severe illness compared to patients without COVID-19 [68].

#### 3.2.2. Subarachnoid Hemorrhage

Subarachnoid hemorrhage is a critical medical condition that involves bleeding into the subarachnoid space, which is located between the arachnoid layer and pia mater that contains CSF [69]. Craen and colleagues reported a case of a subarachnoid hemorrhage in a 66-year-female patient with significant past medical history of diabetes, hypertension, and hyperlipidemia. The patient initially presented with cardiac arrest which resolved spontaneously with cardiopulmonary resuscitation (CPR) and administrations of epinephrine. Prior to the onset of these symptoms, the patient experienced a history of dry cough for one-week, shortness of breath, and general malaise. The patient was intubated after arrival to the emergency department and her chest X-ray showed bilateral infiltrate. The patient was tested for SARS-CoV-2 and was found to be positive. Brain CT scan revealed a subarachnoid hemorrhage extending into the suprasellar cistern, Sylvian and interhemispheric fissures, effacement of the fourth ventricle, and diffuse cerebral edema. The patient was unresponsive, had fixed dilated pupils nonreactive to light, absent corneal and gag reflexes, and absent oculocephalic and vestibulocephalic movements. The patient developed another cardiac arrest and was pronounced dead [70]. Another case of subarachnoid hemorrhage was reported by Al Saiegh and colleagues in a 31-years-old male patient who presented with symptoms of upper respiratory tract infection, mild fever, malaise, cough and arthralgia. The patient developed sudden onset of severe headache and loss of consciousness. A head CT scan revealed a subarachnoid hemorrhage. The patient was intubated and placed on an external ventricular drain. Due to his respiratory complaints, the patient was tested for SARS-CoV-2 and was found to be positive. The patient was extubated on the second day of intubation, his symptoms improved gradually, and he was discharged for rehabilitation [71].

#### 3.2.3. Massive Cerebral Hemorrhage

Massive cerebral hemorrhage is defined as those lesions at least 3 cm in the largest dimension in the cerebral hemispheres or 1.5 cm if the lesion is in the brain stem [72]. A case of massive cerebral hemorrhage in the left temporal lobe, basal ganglia, and radiating coronal area in a 38- year-old man has been reported [73]. The patient presented initially to a local health center with vomiting, changes in consciousness, and falling while having dinner. The patient was transferred to a nearby hospital where he had a brain CT scan which revealed a large amount of cerebral hemorrhage in the left temporal lobe, basal ganglia, and radiating coronal area. The following day, the patient developed difficulty breathing and poor blood oxygen saturation that required endotracheal ventilation. The patient blood tests showed an increase in the neutrophil percentage (91.3%), and decrease in the lymphocyte percentage (2.9%), and high levels of high sensitivity C- reactive protein (CRP) (70.7 mg/L). He underwent a craniotomy to remove the intracerebral hematoma and was transferred to the intensive care unit (ICU). The next day, the patient developed high grade fever of 39.6 °C, and chest CT scan showed patches and strip-like high-density shadows and partial consolidation. Due to the patient symptoms, chest CT scan that showed interstitial inflammation, and lymphocytopenia, the patient was tested for SARS-CoV-2 and was found to be positive [73].

#### 3.2.4. Encephalopathy

Acute encephalopathy is a non-specific term used to describe the acute impairment of brain function which presents clinically as alteration in the level of consciousness [74]. It is mostly triggered by infections, especially those caused by viruses [75]. Many forms of COVID-19- associated encephalopathy have been documented as clinical features of SARS-CoV-2 infection [76]. In a single-center cross-sectional retrospective study by Scullen and colleagues, which included 76 critically ill patients with COVID-19, a total of 23 patients had an evidence of neurological involvement with 74% of patients presented with encephalopathy, and 7% presented with acute necrotizing encephalopathy [76].

Encephalopathy may also present at an early stage of the disease, or as a disease initial symptom. For example, a 72-year-old male patient with significant past medical history presented with symptoms of cough and fever. The patient developed mental status alterations after 24 h of the initial presentation and he was diagnosed with encephalopathy. His electroencephalography (EEG) results showed bilateral slowing and focal slowing in the left temporal region with sharply contoured waves. His meningitis workup was negative, and he later tested positive for COVID-19. He developed a respiratory failure, which required intubation and ICU admission [77]. Additionally, Hosseini and colleagues reported two cases who presented with delirium and changes in mental status as the initial symptoms of COVID-19. The first case was a 46-year-old male patient who developed delirium and confusion after experiencing a headache for two days. The patient laboratory tests, and imaging studies were unremarkable. He was then tested for SARS-CoV-2 infection and the test result was positive. The second case was a 79-year-old female patient who experienced confusion and verbal communication difficulties followed by seizures. The patient tested positive for SARS-CoV-2 infection [78].

Several imaging techniques have been used to detect COVID-19-associated encephalopathy. The results of these studies indicate the development of non-specific changes in patients with COVID-19. For example, the findings from non-contrast CT scan studies indicate the presence of diffuse hypoattenuation of deep white matter and focal hypodensities of deep white and grey matter [76]. Electroencephalogram imaging, a commonly used imaging technique to monitor encephalopathy, revealed patterns specific to COVID-19 patients [79]. A recent study examined the EEG patterns in 20 COVID-19 patients with severe disease in comparison with patients with infectious toxic encephalopathy, and post-cardiorespiratory arrest encephalopathy. The quantified EEG (qEEG) technique, which utilizes mathematical analysis of the bioelectric EEG signals, was used successfully to distinguish these three types of encephalopathy by identifying specific EEG patterns. Although raw EEG data revealed normal physiological patterns in patients with COVID-19, unique encephalopathic patterns with excess generalized delta waves and lower alpha and beta values were identified [79].

In an observational study, Neumann and colleagues analyzed CSF of 30 patients with neurological complications. CSF analysis showed normal or slightly increased white blood cell count (WBC), and most cases showed normal CSF blood albumin ratio. Oligoclonal bands were negative in 14 of 25 tested cases (56.0%). Importantly, SARS-Cov-2 was not detected in CSF of all 30 cases [27]. A retrospective study by Paterson and colleagues reviewed the neurological complications in patients who were referred to the National Hospital, Queen Square COVID-19 multidisciplinary team meeting (COVID-MDT) and found that out of 43 patients, ten patients presented with para- infectious or septic encephalopathy with no CSF abnormalities. They also found that Long-term neurological complications were not dependent on the severity of the respiratory illness [28].

#### 3.2.5. Acute Hemorrhagic Necrotizing Encephalopathy

Acute necrotizing encephalopathy is a rapidly progressive neurologic disorder that usually occurs subsequent to viral infections [80]. A review by Poyiadji and colleagues reported a case of acute hemorrhagic necrotizing encephalopathy. The patient was a female in her 50s who presented with respiratory complaints including cough, and fever in addition to an altered mental status. CSF analysis was found to be negative for bacterial infections and negative for common viral infections. Testing for SARS-CoV-2 infection in the CSF was not performed during the initial assessment. However, the patient was later diagnosed with COVID-19 based on the results of a reverse transcription polymerase chain reaction (RT-PCR) test. The patient’s brain magnetic resonance imaging (MRI) study demonstrated the presence of hyperintensity in the bilateral medial temporal lobes and thalami [32]. Another case of a 59-year-old woman with transfusion-dependent aplastic anemia presented with a seizure and altered consciousness, which preceded the onset of COVID- 19 symptoms by 10 days. The patient was later diagnosed with COVID-19. In addition, lymphocytopenia and severe thrombocytopenia were also confirmed based on the patient’s blood laboratory tests. The results of the imaging studies revealed patterns consistent with a diagnosis of diffuse hemorrhagic acute necrotizing encephalopathy [81].

#### 3.2.6. Encephalitis

Encephalitis is a serious condition with significant impact and burden. About 20–50% of encephalitis cases are caused by viruses, and half of the cases have no known etiology [82]. The disease causes a variety of neurological abnormalities, such as altered consciousness, hallucination, confusion, abnormal movement, and aphasia. MRI Imaging studies of different types of encephalitis may show hemorrhage, enhancement, and restricted diffusion [82]. Ye and colleagues reported a man diagnosed with COVID-19 who had a deterioration in his status and confusion. Further investigation revealed leukopenia, lymphopenia, ground glass appearance based on chest CT, and normal brain CT results. The patient also had symptoms of meningeal disease and a positive Babinski sign. Therefore, CSF analysis was performed and showed no abnormalities, except for elevated CSF pressure. Additional careful neurological evaluation led to a diagnosis of encephalitis. The patient was treated with mannitol, which reduced his CSF pressure and improved in his level of consciousness [83]. Another case of encephalitis was reported in a 41-year-old diabetic woman with COVID-19. The result of her CSF analysis was consistent with a viral infection. She had elevated white blood cell count (70 cells/µL), mostly lymphocytes, high red blood cells count (65 cells/µL), a protein level of 100 mg/dL, and CSF glucose level of 120 mg/dL. She was diagnosed with isolated SARS-CoV-2-induced encephalitis and, interestingly, she had no respiratory symptoms or any other systemic involvement [21,22]. Another COVID-19-associated case of encephalitis was also described, in which the results of MRI imaging were similar to high- grade glial tumor [84].

#### 3.2.7. Anti-N-Methyl-D-Aspartate Receptor (NMDAR) Encephalitis

Patients with anti-NMDAR encephalitis usually present with psychosis, due to the inflammation caused by antibodies specific to NMDARs [85]. Panariello and colleagues reported a case of encephalitis in a 23-year-old male psychiatric patient in the Lombardy Region of Italy. The patient had psychomotor agitation, anxiety, and psychotic features including thought disorganization, persecutory delusions, and auditory hallucinations for three days. The patient was hospitalized and diagnosed with COVID-19 on admission. The patient was medically free, but he is a known illicit drug user since the age of 18 years. The patient was treated with several classes of antipsychotics and intranasal midazolam with no clinical improvements. A CT scan of the chest revealed patchy bi- basilar consolidation. The patient was treated with antibiotics, hydroxychloroquine and darunavir/cobicistat while treatment with antipsychotics was discontinued. Three weeks post-admission, the patient’s neurological symptoms worsened, and he developed severe dysphagia, dyskinesias, and autonomic instabilities. The patient was found to have hyponatremia, increased interleukin 6 (IL-6) levels and CSF anti-NMDAR antibodies. He was diagnosed with anti-NMDAR encephalitis and treated with high-dose dexamethasone and intravenous (IV) immunoglobulin, which improved his clinical status [86]. Although the patient is a known drug user which could make it difficult to conclude that his psychosis symptoms are exclusively due to COVID-19, the presence of anti-NMDA antibodies in his CSF analysis confirms the diagnosis of anti-NMDA encephalitis [87]. Recently, more cases of anti-NMDA encephalitis associated with COVID-19 infection were also reported [88,89].

#### 3.2.8. Meningitis/Encephalitis

The detection of SARS-CoV-2 RNA in the CNS was described in a 24-year-old male patient with meningitis and encephalitis who presented with fever, headache, and generalized fatigue [90]. The patient was diagnosed with influenza at the time of presentation and was treated with laninamivir and antipyretic agents. However, his symptoms worsened over nine days and he developed disturbance in his consciousness and a generalized seizure that lasted for one minute. He had a Glasgow Coma Scale of 6, and experienced neck stiffness. MRI imaging revealed hyperintensity along the wall of the inferior horn of the right lateral ventricle. Although SARS-CoV-2 RNA was detected in his CSF, it was not detected by nasopharyngeal swab. The patient was intubated, mechanically ventilated, and was treated with IV ceftriaxone, vancomycin, acyclovir and steroids [90].

#### 3.2.9. Acute Myelitis

A case of acute myelitis in a 66-year-old male patient with COVID-19 has been reported by Zhao and colleagues. The patient was admitted to the hospital due to complaints of fatigue and fever for two days prior to admission. He tested positive for SARS-CoV-2 infection and his CT scan showed patchy changes in the lungs. The patient continued to have high grade fever of 40 °C and he developed weakness in both of his lower limbs with urinary and bowel incontinence. Sensation in the lower limbs was impaired below the level of T10, however, sensation in the upper limbs remained intact at a level of T10 or higher. CSF analysis and MRI of the spinal cord were not performed due to the ongoing pandemic during hospitalization, but diagnosis of acute myelitis was made based on the clinical findings of the patient assessments. Several drugs were used to treat the patient including antibiotics, antivirals, dexamethasone and human immunoglobulin. The treatment resulted in improvements in the patient clinical status and his temperature and oxygen levels. The muscle strength of his lower limbs was 1/5 and he was discharged and transferred for rehabilitation therapy. These clinical findings may indicate that the patient had a cytokine storm based on the high levels of inflammatory mediators, such as CRP and IL-6, which indicate an overactive inflammatory response mediated by the hyperactivation of the immune system [91].

### 3.3. Demyelinating Disorders

#### 3.3.1. Guillain–Barré Syndrome

The first report of COVID-19-associated Guillain–Barré syndrome to our knowledge was not conclusive. It remains unclear whether the case was a mere coincidence or directly linked to COVID-19 [92]. However, subsequent studies provided compelling evidence for potential association of COVID-19 with Guillain–Barré syndrome. Among 1000–1200 patients with COVID-19 admitted to a hospital in northern Italy, five cases of Guillain–Barré syndrome were reported [93]. Moreover, Finsterer and colleagues reviewed 24 cases of Guillain–Barré syndrome that were potentially associated with COVID-19. Among these cases, male and elderly patients were the most affected, and the majority of the cases occurred subsequent to the onset of COVID-19 symptoms, with a mean latency of 9 days. The predominant disease subtype observed was acute, inflammatory, demyelinating poly-radiculoneuropathy, which accounted for 58% of cases. Notably, CSF analysis was negative for viruses. Nearly 29% of patients required artificial ventilation, and this was due to Guillain–Barré syndrome rather than the severity of COVID-19 infection itself. Generally, patients had a good response to IV immunoglobulin treatment [35].

Additionally, autonomic dysfunction associated with COVID-19-related Guillain–Barré syndrome was also observed in diagnosed patients. For instance, a 72-year-old man with comorbidities came to hospital complaining of symmetrical ascending weakness and paresthesia which was preceded seven days earlier by diarrhea, anorexia, and chills [94]. Interestingly, the patient had no fever or pulmonary symptoms. A panel of investigations were performed, and the patient tested positive for SARS-CoV-2 infection. Chest X-ray result showed mild bibasilar atelectasis versus patchy consolidations. Three days post-admission, he was transferred to the ICU due to worsening respiratory status. The patient was diagnosed with Guillain–Barré syndrome and was treated with IV immunoglobulin. One day later, he developed autonomic dysfunction manifested as alternating hyper/hypo tension, and tachycardia. Then, at day 6 post-admission, he experienced a significant decrease in muscle power and became quadriplegic. Two days later, he developed syndrome of inappropriate antidiuretic hormone secretion [94].

Guillain–Barré syndrome can present also with atypical features [36]. Assini and colleagues documented two cases of such atypical Guillain–Barré syndrome. The first case involved a 55-year-old man who developed Guillain–Barré syndrome/Miller Fisher Syndrome overlap syndrome. The patient was initially admitted to the hospital due to COVID-19, and then, twenty days post-admission, he developed acute bilateral eyelid ptosis, dysphagia, dysphonia, and hyporeflexia in his upper and lower extremities. Oligoclonal bands were present in his CSF and serum, and motor nerve conduction study findings were consistent with Guillain–Barré syndrome. The patient condition improved tremendously following IV immunoglobulin treatment. The other case involved a 60-year-old man who had acute motor sensory axonal neuropathy with dysautonomia. Twenty days following severe pneumonia caused by SARS-CoV-2, he developed acute weakness in his lower extremity and right foot drop along with loss of autonomic function manifested with uncontrolled blood pressure, paralytic ileus and gastroplegia, and he had no deep tendon reflex on physical examination. Oligoclonal bands were present in CSF and serum, and electroneurography revealed findings of severe sensory-motor axonal polyneuropathy. The patient was treated with IV immunoglobulin and his condition improved significantly [36].

#### 3.3.2. Miller Fisher Syndrome

Miller Fisher Syndrome and polyneuritis cranialis, which are considered as variants of Guillain–Barré syndrome, have been reported in two patients with COVID-19 [37]. Both patients had normal CSF analysis results. The first patient was a 50-year-old man who initially experienced fever, cough, headache, malaise, and low back pain in addition to anosmia and ageusia. Five days later, he developed right internuclear ophthalmoparesis, right fascicular oculomotor palsy, and ataxia. The patient also had areflexia on physical examination. Chest CT and X- ray imaging results were normal, but laboratory investigations revealed lymphopenia and elevated CRP. Anti-gangliosides antibodies testing results were negative, except for the antibody GD1b-IgG. He was treated successfully with immunoglobulin, which suggests that his condition was caused by an immune-mediated mechanism. The second patient was a 39-year-old man who complained of diarrhea, low-grade fever, and generalized fatigue. Three days later, he developed acute onset diplopia. Neuro-ophthalmological examination revealed bilateral abducens palsy and absence of deep tendon reflexes. Additional laboratory investigations revealed leukopenia. The patient was discharged and treated with acetaminophen, which resolved all his symptoms [37].

#### 3.3.3. Central Nervous System Demyelination

Demyelinating disorders that were found to be associated with COVID-19 could also affect the CNS [95]. For example, a 54-old-woman was admitted to the hospital due to loss of consciousness at home. Her Glasgow Coma Scale was 12 and she was found to have gustatory and olfactory dysfunction before losing consciousness. Further investigation confirmed SARS-CoV-2 infection. Her status deteriorated and she experienced two seizures confirmed by EEG. Further laboratory and MRI imaging investigations identified new demyelinating lesions in the periventricular white matter, bulbomedullary junction, cervical spinal cord, and dorsal spinal cord [95].

Delayed post-hypoxic necrotizing leukoencephalopathy (DPHL) was also reported to be associated with COVID-19 [96]. A 50-year-old man diagnosed of COVID-19 was first admitted with oxygen saturation of 90% that dropped on the second day to 70% despite receiving supplemental oxygen. The patient had no neurological deficits at the initial presentation, but he developed altered mental status and occasional myoclonic movement. The patient recovered gradually of the pneumonia over two weeks, however, his altered mental status and occasional myoclonic movement persisted. EEG results revealed no epileptiform firing but rather a moderate diffuse slowing. MRI imaging results showed large areas of white matter demyelination with active demyelination and necrosis. These findings are consistent with DPHL [96].

### 3.4. Seizures

Seizures associated with COVID-19 can occur as a sequel of encephalopathy or because of the severe illness associated with non-epileptic seizure without brain injury [97]. In either case, seizures could be the initial presenting manifestation of COVID-19. Vollono and colleagues reported a focal epilepticus as the initial presentation in a patient with COVID-19. The patient was a 78-year-old woman with hypertension and drug- controlled postencephalitic epilepsy. After a seizure-free period of 2 years, she developed a focal epilepticus which was confirmed by EEG. MRI imaging showed a large area of gliosis and left temporo-parietal lobe atrophy caused by her past medical condition. She tested positive for SARS-CoV-2 and the results of her laboratory investigations showed lymphopenia, thrombocytopenia, and elevated CRP. The status epilepticus was managed by antiepileptics and the patient was discharged 16 days after admission [98].

Non-epileptic seizures were also reported as the initial presentation for COVID-19 [99]. A 70-year-old woman had many episodes of seizures and syncope and was unconscious on admission. Her vital signs were normal, and no upper respiratory, cardiopulmonary, and other neurological symptoms were observed. MRI results revealed no acute lesions, and the results of EEG and CSF analysis were normal, but further laboratory investigations showed lymphopenia and elevated CRP. She then developed dyspnea with oxygen saturation of 82% and tested positive for SARS-CoV-2. The patient also had autonomic dysfunction as indicated by sympathetic skin response, which might have been aggravated by COVID-19 [99].

### 3.5. Symmetrical Polyneuropathy

A case of symmetrical polyneuropathy with COVID-19 has been recently reported [100]. A 68-year-old woman presented initially to the hospital with dry cough, fever, and myalgia over the last three days. The patient had a medical history of diabetes mellitus, end-stage renal disease, and rheumatoid arthritis. Her temperature was 39.9 °C, and her chest CT scan showed bilateral patchy high-density shadows. Blood tests showed lymphopenia, hyponatremia, elevated creatinine, normal erythrocyte sedimentation rate (ESR), high CRP and high blood glucose level at time of admission. The patient SARS-CoV-2 test result was positive, and she was treated with oxygen supplementation, lopinavir/ritonavir, and oseltamivir. On the third day of admission, she developed bilateral weakness of lower extremities, and her deep tendon reflexes were absent. Neurological examination and cranial nerve examination were unremarkable. The patient was started on methylprednisolone due to suspicion of a virus-related immune reaction. On the sixth day of admission, the patient developed difficulty breathing and her oxygen saturation at that time dropped to 78%. She was intubated and mechanically ventilated. CT scan of the chest showed severe bilateral ground-glass opacities. Based on these finding, the patient was diagnosed with acute ARDS. The patient experienced cardiac arrest and died despite several CPR attempts [100].

### 3.6. Rhabdomyolysis

Gefen and colleagues reported a case of a 16-year-old boy with COVID-19 and rhabdomyolysis. The patient had a medical history of autism spectrum disorder, attention deficit hyperactivity disorder, morbid obesity, obstructive sleep apnea, and eczema. He presented with a history of fever lasting for one day, myalgia, and shortness of breath with exertion for 4 days, and dark-colored urine for 2 days. The patient blood tests showed elevation in aspartate transaminase (AST) level (839 U/L), alanine aminotransferase (ALT) level (157 U/L), and creatine kinase (CK) level (427,656 U/L). Urinalysis revealed blood in the urine with 11–25 RBC/HPF and 6–10 WBC/HPF. The patient was tested for COVID-19 and the result was positive. He received IV fluids containing sodium bicarbonate and potassium chloride. The CK levels improved the following days but the myalgia did not improve, which required treatment with acetaminophen. The IV fluids were discontinued after 11 days, and the patient was discharged at day 12 with a CK level of 6526 U/L [101].

## 4. COVID-19 Associated Neurological Symptoms in Pediatrics

Although studies have shown that children experience mild forms of COVID-19 disease, rare neurological complications have been reported. Dugue and colleagues reported a child with COVID-19 who had dystonic leg stiffness, abnormal gaze, and brief alteration in responsiveness [102]. Another case was reported for a child with COVID-19 who developed viral encephalitis. The condition was reversible, and the patient achieved full recovery [103]. Neurological complications of COVID-19 seem to be higher in children with multisystem inflammatory syndrome (MIS-C) [104]. Reviewing 187 children with MIS-C from six studies showed a high incidence of neurological complications in MIS-C patients with COVID-19. Neurological manifestations included headache, meningism and mental status alteration. Interestingly, most patients achieved full recovery with IV immunoglobulins or steroid treatment [104]. In another study, Abdel-mannan and colleagues reported four cases of MIS-C with COVID-19 neurological complications. Radiological imaging in those patients showed splenium signal changes [105]. Another recent international multicenter study analyzed neuroimages of 38 children with COVID-19 related neurological complications and found that the most common neuroimaging abnormality was para infectious immune-mediated. Acute disseminated encephalomyelitis-like changes were described in 16 patients. Other abnormalities include myelitis in eight patients, neural enhancement in 13 patients, splenial lesions in seven patients and myositis in four patients. Splenial lesions and myositis were predominant findings in patients with MIS-C. Evidence for thromboembolic or vasculitic findings were also present in 18% of the patients [106].

## 5. Neurological Side Effects Associated with COVID-19 Treatments

Since the early onset of the pandemic, several treatments have been tested and developed to treat COVID-19. These treatments have reduced the severity of the disease and the time patients spend at the hospital. However, many of those treatments that are being used for COVID-19 have the potential to cause neurological or psychiatric symptoms [107]. An example of these medications is lopinavir-ritonavir combination treatment which was found to be associated with bilateral sensorineural hearing loss [107]. Dexamethasone which has emerged as an effective treatment that reduces mortality in COVID-19 patients who were admitted to the ICU is also associated with many side effects [108,109]. Corticosteroids inhibit the immune response in patients and modulate the hyperinflammation which is critical for the host to induce the immune response [108]. Although it is more common in patients receiving long-term corticosteroids treatment, neuropsychiatric symptoms are common side effects [109]. Other side effects that were observed in patients receiving corticosteroids include memory deficits and cognitive impairment [109]. The side effects of corticosteroids depend on several factors including the route of administration, the dose and the duration of treatment [109]. On the other hand, erythropoietin (EPO) which has emerged as a supportive treatment for patients with severe COVID-19, has a neuroprotective and neuro-regenerative effects [110].

## 6. Post-Acute COVID-19 Syndrome

Post-acute COVID-19 syndrome is a term that has been used recently to describe the complications that extend beyond the duration of initial illness and after recovery of SARS-CoV-2 infection [111]. Recently, those complications have becoming a well-recognized status which has been suggested as a post-infectious entity [111]. Fatigue, changes in concentration, loss of memory, sleep disorders, cough and dyspnea were the main reported symptoms [111]. These complications include mainly neurological symptoms which could be a persistence of some symptoms or appearance of new symptoms after the recovery [111]. A report from the CDC indicated that 35% of patients with mild COVID-19 did not return to the baseline after recovery [112]. A study by Tabacof and colleagues investigated the post-acute COVID-19 symptoms that persist for more than six weeks after the onset of acute symptoms and found that the most persistent symptoms among patients were fatigue (92%), loss of concentration or memory (74%), weakness (68%), headache (65%) and dizziness (64%) [111]. Another study by Carfi and colleagues found that 87.4% of COVID-19 patients reported the persistence of at least one symptom, with fatigue being the most common reported symptom, followed by dyspnea. Importantly, the quality of life was affected in 44.1% of the patients [113]. Another prospective cohort study by Moreno-Perez and colleagues evaluated the incidence of post-acute COVID-19 syndrome in adult patients. The patients were evaluated with a systematic assessment for 10–14 weeks after the disease onset. The study detected post-acute COVID-19 syndrome in half of COVID-19 survivors and the persistence symptoms in those patients were mostly mild [114].

## 7. Conclusions

COVID-19 has had a significant devastating impact on the world. SARS-CoV-2 has infected millions of people and caused thousands of deaths, and each day, we are learning and developing better understanding of this novel virus. According to many studies, COVID-19 can cause both acute and long-term neurological complications in many patients. Although there is hope with vaccine developments to contain the pandemic, it is critical to further investigate and understand the clinical manifestations of COVID-19 and mechanisms. This will broaden our knowledge and help in studying the pathogenesis of this novel disease, which could lead to the development of new treatments.

## Data Availability

Data sharing is not applicable to this article

## Abbreviations

COVID-19	coronavirus disease 19
SARS-CoV2	Severe acute respiratory syndrome-coronavirus 2
CNS	central nervous system
PNS	peripheral nervous system
CPR	cardiopulmonary resuscitation
NMDA	N-Methyl-D-aspartic acid
NMDAR	N-Methyl-D-aspartic acid receptor
IL-6	Interleukin 6
CRP	C-reactive protein
AST	Aspartate transaminase
ALT	Alanine transaminase
CK	Creatine Kinase
ACE2	Angiotensin-converting enzyme 2
PCR	Polymerase chain reaction
ARDS	Acute Respiratory Distress Syndrome
WHO	World Health Organization
IgM	Immunoglobulin M
EEG	Electroencephalography
MRI	Magnetic resonance imaging
EPO	Erythropoietin
CSF	Cerebrospinal fluid
HSP	human heat shock proteins family
